# Remarks on the Pathology and Treatment of Stricture of the Urethra*Read before the Medical Society of London.

**Published:** 1871

**Authors:** W. F. Teevan

**Affiliations:** Surgeon to the West London Hospital and to St. Peter’s Hospital, late Lecturer on Anatomy at the Westminster Hospital


*Remarks on the Pathology and Treatment of Stricture of the Urethra.**
By W. F. Teevan, B.A., F.R.C.S., Surgeon to the West London Hospital and to St. Peter's Hospital, late Lecturer on Anatomy at the Westminster Hospital.

---

I intend here to make a few very brief remarks on the pathology and treatment of stricture of the urethra, as time will not permit me to do more than merely enunciate a series of facts which I have by inquiry ascertained. It appears to me that, in order to arrive at any satisfactory results, we must be thoroughly acquainted with certain facts required to be elicited; next, see what are the indications as to a scientific treatment; and, lastly, we must find out the values of the varying remedial agents which we propose to ourselves as means of cure. I am constantly in the habit of putting to students who have undergone a professedly thorough course of surgical training, this simple question: When would you say that a man was suffering from stricture of the urethra?—and I rarely get a correct answer. Students have but a vague and unsatisfactory knowledge of the pathological state. How, indeed, could it be otherwise?—for most surgeons would declare that if a No. 10 English catheter could be passed into a man's bladder, there could be no stricture; and we thus have this most extraordinary and serious fact presented that the urethra may dwindle down to one-third its natural calibre, without so grave a pathological state being recognized—nay, I would say, not even suspected. I consider that the diagnostic power of an educated surgeon ought to be something more and above the untutored perception of a working man; but in this complaint it is not so, for it usually happens that the patient is the first to discover the existence of a stricture by informing us that he has a difficulty in making water. Not until the enemy is at the door is the surgeon aware of his presence; scout or skirmisher he hath not. It ought to be our aim to recognize the disease in its earliest incipiency, and so abolish all necessity for any recourse to operative procedures. Can we do so? Most certainly. If there be one complaint which more than another worries a patient, whether he be rich or poor, it is the continuous presence of a gleet; and for this disease he goes about from hospital to hospital, seeking to get it cured. Now, if such a patient present himself to us with a gleet of more than six months' duration, we shall always, on examining the urethra, find important pathological alterations, the most serious of which is contraction. The only instrument which can effect the acquirement of the knowledge we seek is the bougie a boule. I define stricture to be "any diminution of the natural calibre of the urethra, the result of the contraction of organized lymph." This definition, consequently, embraces the disease in all its stages; and if we diagnose the complaint in its earliest incipiency, we shall render the sufferer

*Read before the Medical Society of London.

an immense service, for we shall entirely purge the disease of its evil effects on his renal organs, and abolish all operative procedures. The first sign of a stricture is the presence of a gleet of more than six months' duration; and, long before we have the symptoms resulting from a mechanical contraction, we have usually some more or less well-defined symptoms, such as increased micturition, pain in the loins or perineum, trembling at the knees whilst urinating, or a sensation of cold water running down the spine. I would, however, insist upon one point, and that is, whenever a patient comes to us complaining of a gleet of some months' duration, we ought never to omit to examine the urethra with the ball-staff.

Now, a few words as to the seats and physical attributes of strictures. Let it be ever kept in mind that stricture is atrophy, not hypertrophy; and that not only does the urethra become contracted, but also torturous, which latter fact shows the inapplicability of metal instruments in the treatment of the disease. Strictures are usually situated in the triangular ligament, and their division into bulbous and membranous is entirely artificial, and not warranted by any facts; I group these together, and call them alike "subpubic," thus simplifying the nomenclature. It will be found that this stricture is usually at a spot five inches and a half from the meatus externus. Why is this variety of stricture the most common? For the simple reason that, when there is increased vascularity between the canal and the ligament, the latter commences to act as an irritant and constricting agent by preventing the expansion of the urethra in the act of micturition. But you may say to me—How, then, is it we sometimes get a penile stricture without a subpubic one? For the reason that we constantly meet with men whose fibrous apertures are larger than they ought to be. If the aperture in the triangular ligament be larger than the normal size, the edges of the opening will not exercise any constriction on the urethra when expanded in the act of micturition. If, therefore, we meet with penile stricture only, we may conclude that it is besause the patient has a very large aperture in his triangular fascia. If we examine one hundred patients, we shall find that eighty have the subpubic form of stricture; eighteen the penile variety, which is situated at a spot varying from $2\frac{1}{2}$ in. to $3\frac{1}{2}$ in. from the meatus externus; and the remaining two have strictures at the orifice, which I consequently call orificial. It is rare to meet with a subpubic stricture without finding some induration or leatheriness at the penile spot. Orificial strictures are often caused by chancrous ulceration. I have measured the urethræ of one hundred adult males, and find the average length to be $7\frac{1}{8}$ in., thus rather shorter than is usually supposed. Strictures give to the bougie a boule either the sensation of traveling through a *tunnel*, or the feeling of passing through a sharp and well-defined *ring*. I therefore speak of strictures as being either tunnel or ring strictures. Subpubic strictures are generally of the former kind, whilst orificial are of the latter. Penile strictures are as often ring-like as tunnel-like.

What is the result required to effect a cure of a stricture? No urethra can ever be said to be cured till the diseased and contracted portion is restored to its normal calibre. Some time ago I instituted a series of practical inquiries to ascertain what was the normal capacity of the urethra, and I found that the subpubic urethra will admit the terminal joint of the fore-finger without laceration. The results will be found at p. 186 of the "Pathological Society's Transactions" for 1866. Now, all means which have not for their end the restoration of the urethra to its normal calibre will fail—that is to say, if the urethra, by dilatation, sudden or gradual, rupture, or incision, be only enlarged to a diameter less than its normal capacity, we shall most infallibly have a return of the contraction, unless we from time to keep up the diameter of the tube to that which we had stretched it. In severe forms of stricture there is only one instrument that can be introduced, and that is the French filiform bougie; and I venture to assert that, with patience and perseverance, a surgeon will rarely fail to introduce this instrument. Whatever treatment be adopted for stricture, it must always be followed, and nearly always preceded, by gradual dilatation. It thus appears that there is *only one* which can be called *the treatment*—that by gradual dilatation; all other methods are merely auxiliaries. The bougie can nearly always dispense with the knife or dilator, but they are useless without the bougie. In a limited number of cases the bougie will fail to enlarge the contracted urethra beyond a certain point, or if dilated to a considerable size, it will speedily contract again. In such cases an operation is desirable.

All operations on the urethra may be divided into two classes: those which burst the stricture by lacerating, and those which divide it by cutting. Which are preferable? Surgical pathology settles the point at once, for we know that there is more contraction after a laceration than after a cut. Hence we ought to choose that kind of operation which is attended with the least contraction, which, consequently, will be the cutting one. From what I have seen of Mr. Syme's operation in the practice of different surgeons, and from all I have heard of it as practiced by its originator, I have come to the conclusion that it ought never to be resorted to unless the state of the perineum is such as to preclude all other operations. The treatment of stricture by forcible rupture would seem in this country to be following a similar fate to what it had in Paris, where it was first introduced, as it has been discontinued by Sir W. Ferguson, Mr. Coulson, Mr. Henry Smith, and other surgeons. The unknown rate of mortality attached to it is not calculated to recommend it, and I am aware of twenty-one deaths which have followed its use in the hands of different hospital surgeons. The great advantages which gradual dilatation, when carried out by the French elastic instruments, possesses over all other methods is, that it is absolutely harmless, no death ever having taken place from its employment, and that it can be employed in the most diseased subject without the slightest fear. It is also the only treatment which can restore the urethra to its normal calibre.

But whatever operation be employed for the relief of stricture, it must be supplemented by the regular use of the bougie for the rest of the patient's life, as no known method of treatment is free from an infallible relapse apart from its use. It follows, therefore, that, inasmuch as in the majority of strictures the bougie can effect all, and more than the knife or dilator can do, and, unlike them, can never kill a patient, it presents itself to us for our acceptance with recommendations possessed by no other known instrument; and I venture to assert that the bougie will be used when urethrotomes and dilators are forgotten.

The treatment of stricture may be summed up—1. Gradual dilatation, wherever possible. 2. Subcutaneous urethrotomy, wherever desirable. 3. External urethrotomy, without a guide, wherever necessitated.—*Lancet.*

